# Recurrent Angina in Apical Hypertrophic Cardiomyopathy Associated With Myocardial Bridging of the Posterior Descending Artery

**DOI:** 10.7759/cureus.111722

**Published:** 2026-06-29

**Authors:** Mihir G Shah, Yashvardhan Batta, Carly Fabrizio

**Affiliations:** 1 Internal Medicine, Temple University Hospital, Philadelphia, USA; 2 Cardiology, Temple University Hospital, Philadelphia, USA

**Keywords:** angina, cardiac events, coronary angiography, hypertrophic cardiomyopathy, myocardial bridge, posterior descending artery

## Abstract

Chest pain with apical hypertrophic cardiomyopathy (HCM) warrants concern for acute coronary syndrome (ACS). When obstructive coronary disease is excluded, myocardial bridging should remain an important consideration. We report a 60-year-old female with apical HCM and multiple prior evaluations for a mix of typical and atypical chest pain who presented with worsening dyspnea on exertion and stable intermittent chest pain. Coronary angiography revealed a myocardial bridge involving the posterior descending artery (PDA). Given the increased prevalence of myocardial bridging in the HCM population and its association with increased risk of myocardial ischemia, workup for angina-like symptoms should incorporate evaluation for a myocardial bridge.

## Introduction

Myocardial bridging (MB) is a congenital coronary artery anomaly characterized by an intramyocardial course of a segment of an epicardial coronary artery, most commonly involving the left anterior descending (LAD) artery, wherein the vessel traverses the myocardium rather than remaining on the epicardial surface [1]. Although frequently considered a benign variant in the general population, MB can be associated with clinically significant sequelae, including exertional angina, ventricular arrhythmias, myocardial ischemia, and, rarely, sudden cardiac death [2-5].

In patients with hypertrophic cardiomyopathy (HCM), MB may further exacerbate myocardial ischemia due to dynamic systolic compression and impaired coronary perfusion, especially in the setting of increased myocardial mass and diastolic dysfunction. Given the heightened susceptibility of arrhythmia and ischemia in this population, this case aims to highlight that evaluation for concomitant MB is warranted in HCM patients presenting with angina-like symptoms. We present a case of atypical chest pain in a patient with apical HCM caused by a myocardial bridge in the posterior descending artery (PDA).

## Case presentation

A 60-year-old female with a history of apical HCM, hyperlipidemia, and chronic autoimmune thyroiditis presented to the emergency department with persistent chest pain at rest radiating to her left arm. She had a family history of HCM in her father and sister, and no history of tobacco, alcohol, or illicit drug use. Her current medications included metoprolol succinate, levothyroxine, rosuvastatin, and ezetimibe. On admission, she was 155 cm tall, weighed 55 kg, and had a blood pressure of 140/81 mmHg.

Two days prior to presentation, she was evaluated by a cardiologist for intermittent chest pain described as substernal heaviness radiating to her left arm with accompanying progressive dyspnea on exertion. Given the symptom burden, concern for heart failure, and family history of coronary artery disease, she was scheduled for a right and left heart catheterization. Right heart catheterization hemodynamics demonstrated normal right atrial pressure, pulmonary capillary wedge pressure, pulmonary vascular resistance, cardiac output, cardiac index, moderately elevated systemic vascular resistance, and mildly elevated pulmonary artery pressure (RA, 5 mmHg; PA, 35/15 (22) mmHg; PCWP, 10 mmHg; CO/CI, 4.08 (L/min)/2.7 (L/min/m^2^); PVR, 2.94 WU; SVR, 1647 dsc-5).​​​​​​​

Her medical history was notable for multiple prior outpatient evaluations for both typical and atypical chest pain. She underwent several diagnostic tests, including a pharmacologic nuclear stress test performed four years earlier, which was unremarkable. The pharmacologic nuclear stress test showed homogeneous radiotracer uptake throughout the myocardium on both rest and stress imaging, with normal left ventricular size, no evidence of transient ischemic dilation (0.69), and normal wall motion on gated single-photon emission computed tomography (SPECT) imaging. Cardiac magnetic resonance imaging performed two years earlier demonstrated preserved biventricular systolic function with heterogeneous patchy delayed hyperenhancement consistent with apical HCM. The maximum wall thickness was 15 mm at the apical septum. There was no evidence of an apical aneurysm or cavity obliteration. Late gadolinium enhancement (LGE) quantified as 4.6%, with no dynamic obstruction or provocable left ventricular outflow tract. Diastolic parameters were normal (E/A ratio, 1.15; deceleration time, 176 ms). Cardiac computed tomography performed 18 months earlier revealed a coronary artery calcium score of 0. One year before the current presentation, she was started on metoprolol succinate 25 mg daily, and transthoracic echocardiography showed local apical hypertrophy with preserved systolic function (ejection fraction, 60%-65%).

**Figure 1:**
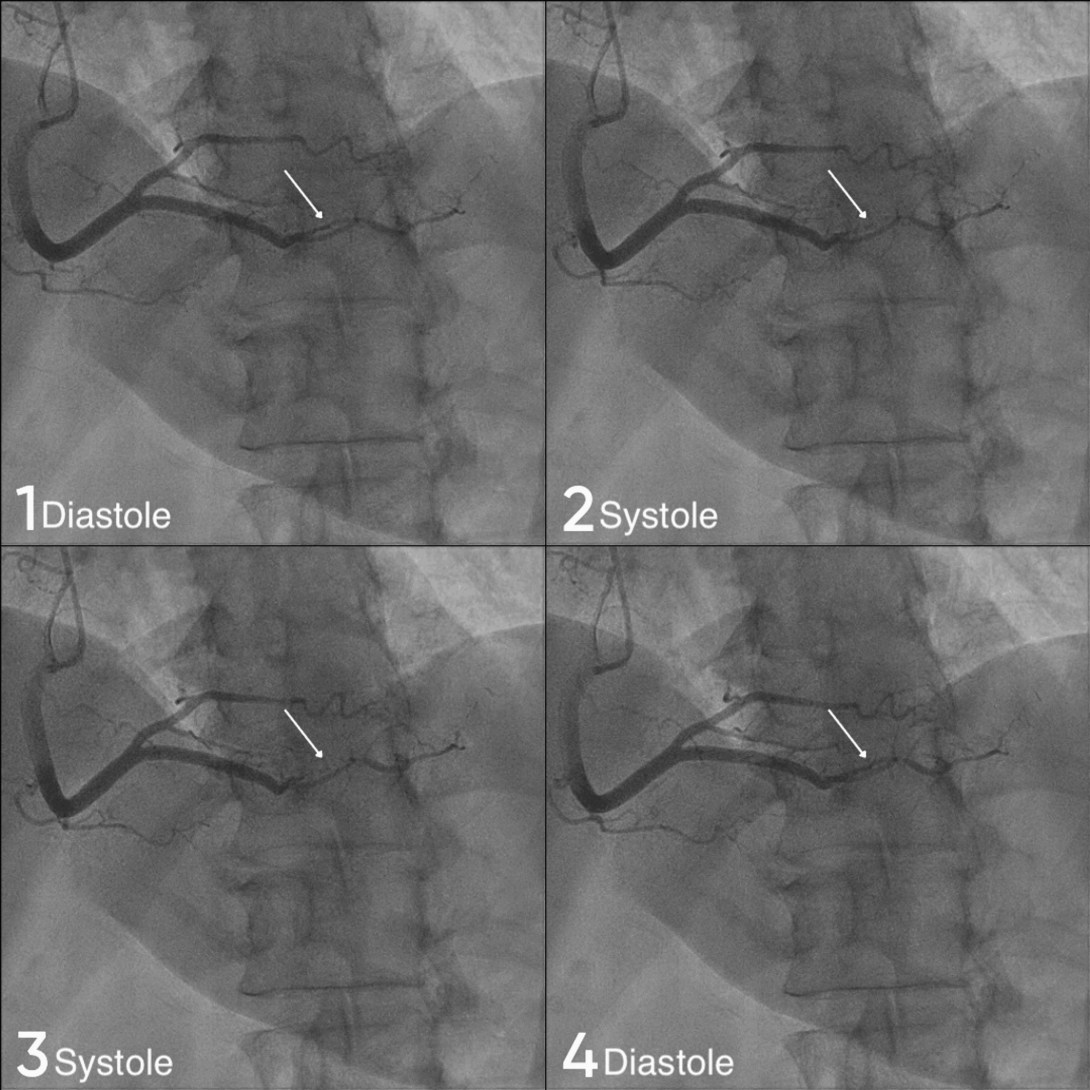
Coronary angiogram showing myocardial bridge of the posterior descending artery in diastole and systole Coronary angiograms in the left anterior oblique (LAO) 23 and cranial 20. The arrow indicates a myocardial bridge of the posterior descending artery during diastole and systole, demonstrating 90% compression.

In light of the current presentation and in the context of apical HCM and recurrent chest pain, the differential diagnosis included non-ST-elevation myocardial infarction (NSTEMI), unstable angina, apical aneurysm, prolonged apical contraction with impaired filling, microvascular dysfunction, and MB. While the patient may have had a combination of diagnoses contributing, she immediately underwent left heart catheterization, which revealed a significant myocardial bridge involving the mid-to-distal PDA (Figure 1). Catheterization revealed right coronary artery dominance with 30% ostial stenosis in the first obtuse marginal artery. The bridge was measured to be 20 mm in length with 90% of systolic compression and no persistence of diastolic narrowing. The instantaneous wave-free ratio (iFR) was not performed because the outflow vessel after the bridged segment was a small territory. Post catheterization, the patient’s metoprolol succinate was uptitrated to 50 mg. The patient was never prescribed a nitrate that could have worsened symptoms, given that the vasodilation of the coronary arteries increased compression of the artery, worsening angina. On follow-up four weeks later, she reported improvement in angina-like symptoms and improved dyspnea on exertion.

## Discussion

We present a patient with established apical HCM presenting with recurrent episodes of chest pain over several years, with subsequent progression to exertional dyspnea and substernal discomfort at rest. Notably, her symptoms persisted despite noninvasive imaging and beta-blockade treatment, prompting further invasive assessment.

MB is a congenital coronary anomaly in which an epicardial coronary artery courses intramyocardially and undergoes dynamic systolic compression [6]. Although MB most commonly involves the mid-LAD artery, bridges affecting other coronary territories have been described and may be clinically relevant, particularly in specific disease states such as HCM [7]. To our knowledge, MB involving the PDA in association with apical HCM has been rarely reported in the literature. This case underscores the importance of considering MB in the differential diagnosis when a patient with apical HCM presents with chest pain, even when symptoms are atypical, and the involved vessel is non-LAD.

MB is more prevalent than traditionally appreciated. In the general population, its estimated prevalence approaches 18% via coronary computed tomography angiography, with rates increasing to 37% via autopsy in the population with HCM [8]. The pathophysiologic significance of MB in HCM stems from the additive ischemic burden it creates in an already vulnerable myocardium. Patients with HCM are inherently susceptible to myocardial ischemia due to multiple mechanisms, including myocardial hypertrophy, microvascular dysfunction with impaired coronary flow reserve, medial hypertrophy and reduced density of intramural arterioles, hyperdynamic systolic function, and left ventricular outflow tract obstruction with high intracavitary pressures [9].

A systematic review and meta-analysis of 10 observational studies found that MB was associated with myocardial ischemia (OR 1.89, 95% CI 1.03-3.44, p = 0.04) but not with cardiovascular mortality (OR 1.70, 95% CI 0.56-5.15, p = 0.35) or nonfatal adverse cardiac events (OR 1.80, 95% CI 0.98-3.28, p = 0.06) [10]. In a retrospective study with 213 patients diagnosed with apical HCM, the MB group had higher rates of chest pain (63.2% vs. 40%; p = 0.003) and were more likely to be in a New York Heart Association (NYHA) class III/IV (14.7% vs. 4%; p = 0.01) [11].

Diagnostic imaging has evolved as invasive and noninvasive methods are available to assess anatomic and functional aspects. Coronary angiography is the traditional method, as it can identify the classic systolic narrowing of the vessel. Within the realm of angiography, there are various intracoronary methods that offer more information, such as vessel wall morphology, atherosclerotic lesions, or flow patterns. Coronary computed tomography angiography (CCTA) provides high-resolution, noninvasive anatomic visualization of the tunneled coronary segment, including its depth, length, and relationship to surrounding myocardium, and therefore identifies myocardial bridges more frequently than angiography. While CCTA excels at defining structure, it is limited in assessing dynamic compression, as image acquisition is typically performed in diastole. Because the clinical relevance of MB is often determined by its impact on coronary flow rather than its mere presence, functional imaging and physiologic testing are increasingly emphasized [1]. Other noninvasive techniques, such as cardiovascular magnetic resonance (CMR), myocardial perfusion imaging, or stress echocardiography, can be useful through physiological assessment of the functional effect, though little anatomic value is extracted. In our case, coronary angiography was performed, given the underlying concern for possible coronary disease contributing to symptoms. CCTA was not performed due to the inability to perform the scan on time.

Importantly, a high index of clinical suspicion is warranted, particularly in patients with HCM or persistent angina-like symptoms despite negative noninvasive testing. Early and comprehensive evaluation at the time of cardiology referral, or even earlier in the diagnostic pathway, may facilitate the timely identification of hemodynamically significant MB and prevent progression to more serious events. In our patient, multiple prior evaluations failed to identify the underlying anomaly, underscoring the need for heightened awareness and proactive diagnostic consideration before complications occur.

## Conclusions

Angina in HCM patients warrants a broad and systematic differential diagnosis. MB should remain in the differential given its increased prevalence and potential cardiovascular consequences, such as ischemia. Although noninvasive testing with CCTA remains the gold standard, and CCTA was not performed in this case, multiple other noninvasive imaging modalities did not identify the myocardial bridge. While there are no guidelines to suggest when to pursue invasive workup, it is the clinician’s responsibility to recognize the potential cause of angina coming from MB. Delay in diagnosis or medical therapies of angina may lead to worsening symptoms, as demonstrated by nitrates in myocardial bridge patients. Clinical history and diagnostic imaging are crucial to diagnosing a myocardial bridge. Importantly, maintaining early clinical suspicion and pursuing timely evaluation may prevent missed diagnoses and reduce the risk of avoidable ischemic complications.
